# Predicting outcomes in older ED patients with influenza in real time using a big data-driven and machine learning approach to the hospital information system

**DOI:** 10.1186/s12877-021-02229-3

**Published:** 2021-04-27

**Authors:** Tian-Hoe Tan, Chien-Chin Hsu, Chia-Jung Chen, Shu-Lien Hsu, Tzu-Lan Liu, Hung-Jung Lin, Jhi-Joung Wang, Chung-Feng Liu, Chien-Cheng Huang

**Affiliations:** 1 Department of Emergency Medicine, Chi Mei Medical Center, 901 Zhonghua Road, Yongkang District, Tainan City, 710 Taiwan; 2 Department of Biotechnology, Southern Taiwan University of Science and Technology, Tainan, Taiwan; 3 Information Systems, Chi Mei Medical Center, Tainan, Taiwan; 4 Department of Nursing, Chi Mei Medical Center, Tainan, Taiwan; 5 Department of Emergency Medicine, Taipei Medical University, Taipei, Taiwan; 6 Department of Medical Research, Chi Mei Medical Center, Tainan, Taiwan; 7 Allied AI Biomed Center, Southern Taiwan University of Science and Technology, Tainan, Taiwan; 8 Department of Senior Services, Southern Taiwan University of Science and Technology, Tainan, Taiwan; 9 Department of Environmental and Occupational Health, College of Medicine, National Cheng Kung University, Tainan, Taiwan

**Keywords:** Emergency department, Influenza, Hospital information system, Machine learning, Mortality, Older, Prediction, Random forest

## Abstract

**Background:**

Predicting outcomes in older patients with influenza in the emergency department (ED) by machine learning (ML) has never been implemented. Therefore, we conducted this study to clarify the clinical utility of implementing ML.

**Methods:**

We recruited 5508 older ED patients (≥65 years old) in three hospitals between 2009 and 2018. Patients were randomized into a 70%/30% split for model training and testing. Using 10 clinical variables from their electronic health records, a prediction model using the synthetic minority oversampling technique preprocessing algorithm was constructed to predict five outcomes.

**Results:**

The best areas under the curves of predicting outcomes were: random forest model for hospitalization (0.840), pneumonia (0.765), and sepsis or septic shock (0.857), XGBoost for intensive care unit admission (0.902), and logistic regression for in-hospital mortality (0.889) in the testing data. The predictive model was further applied in the hospital information system to assist physicians’ decisions in real time.

**Conclusions:**

ML is a promising way to assist physicians in predicting outcomes in older ED patients with influenza in real time. Evaluations of the effectiveness and impact are needed in the future.

**Supplementary Information:**

The online version contains supplementary material available at 10.1186/s12877-021-02229-3.

## Background

The rapidly aging population is one of the most important issues worldwide. In the United States, older adults (≥65 years old) were 15.2% of the total population in 2016, are projected to be 20% in 2030, and 23.5% in 2060 [1]. Taiwan is one of the rapidly aging countries in the world. In 2018, the number of deaths was nearly equal to that of births [2]. Older adults represented 14% of the total population in 2018, and are projected to be 20% in 2025 [2].

Influenza is a life-threatening disease for the older population. An Asian study revealed that older adults contributed to 70–90% of total deaths [3]. The mortality rate of influenza in older adults was nearly 39-fold that of the population aged 40–64 years old [3]. Influenza-related complications, including cardiorespiratory diseases, pneumonia, chronic obstructive pulmonary disease (COPD), and ischemic heart diseases, are the common causes of death [3].

Because of limited medical resources during the influenza season, predicting outcomes in older adults with influenza and their subsequent disposition becomes a critical issue. In our previous study, we recruited 409 older patients with influenza for developing a Geriatric Influenza Death Score (GID score) [4]. This study identified five independent mortality predictors: severe coma (Glasgow Coma Scale [GCS] ≤8), past histories of malignancy and coronary artery disease (CAD), elevated C-reactive protein (CRP) levels (> 10 mg/dl), and bandemia (> 10% band cells) [4]. Three mortality risk and disposition groups were formed according to five predictors: (1) low risk (1.1%; 95% confidence interval [CI], 0.5–3.0%); (2) moderate risk (16.7, 95% CI, 9.3–28.0%); and (3) high risk (40, 95% CI, 19.8–64.2%). The GID score has an area under the receiver operating characteristic curve of 0.86, and Hosmer-Lemeshow goodness of fit of 0.578 [4].

Although the GID score is a potentially good clinical decision rule (CDR) in older adults with influenza, it has the limitations of the small size of derivation sample and lacks both automation and feedback in real time to clinicians [5]. Artificial intelligence (AI) is defined as that uses computer techniques, including machine learning (ML) and deep learning (DL) to represent intelligent behavior [6]. In recent years, a great deal of evidence showed that AI could handle more variables that are already available through electronic health records (EMRs) and may better predict patient outcomes [5]. We performed searches on Google Scholar and PubMed using the keywords “AI,” “death,” “influenza,” “machine learning,” “mortality,” “older adult,” and “outcome,” but we did not find any AI application in this field. Therefore, we conducted the present study for clarifying the issue and applying it in the hospital information system (HIS) to assist decision making in real time.

## Methods

### Study design, setting, and participants

**Fig. 1:**
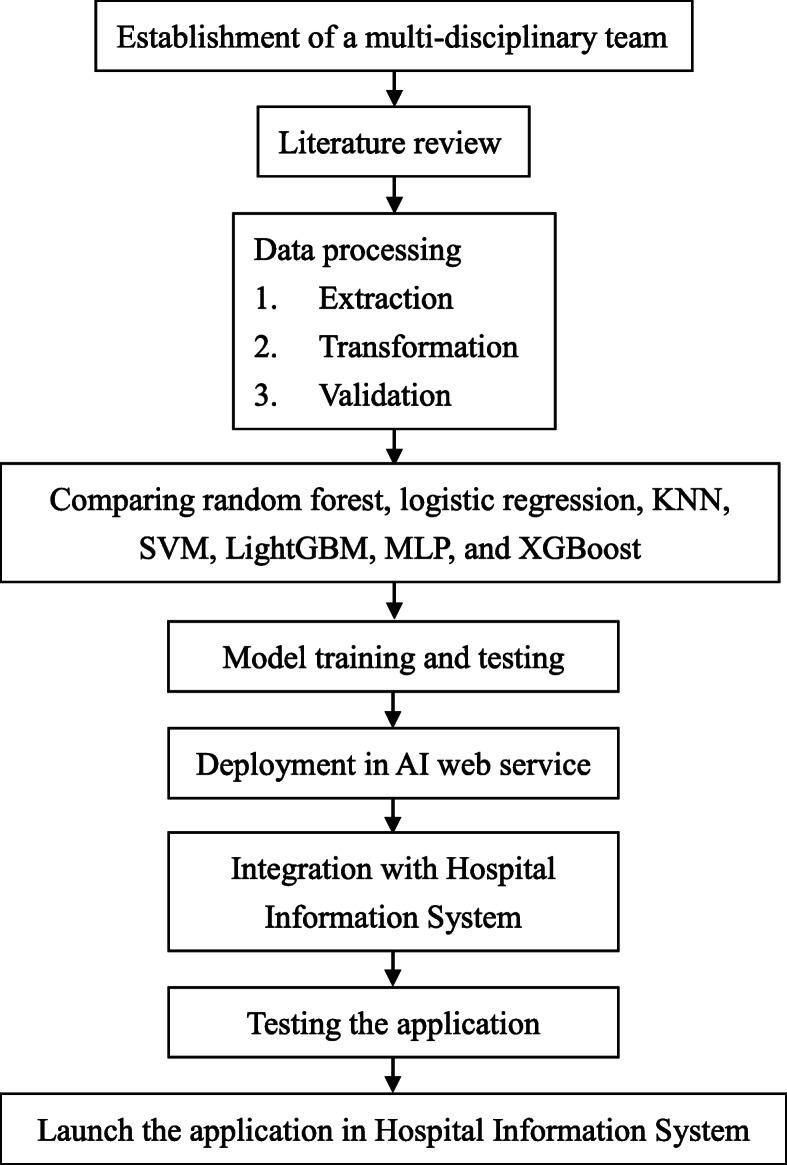
Flowchart of the application of ML for predicting outcomes in older ED patients with influenza. ED, emergency department; KNN, K-nearest neighbors; SVM, support vector machine; LightGBM, light gradient boosting machine; MLP, multilayer perceptron; XGBoost, Extreme Gradient Boosting; AI, artificial intelligence

We included emergency physicians, information engineers, data scientists, quality managers, and nurse practitioners to establish a multi-disciplinary team for this project (Fig. 1). After our literature review, we decided to use the previous study about predicting mortality in older ED patients with influenza as the main reference [4]. We identified all older patients (≥65 years old) with influenza who visited the ED between January 1, 2009, and December 31, 2018, from the EMRs of three hospitals: Chi Mei Medical Center, Chi Mei Hospital, Liouying, and Chi Mei Hospital, Chiali. The present study hospitals are not the hospitals for developing the GID score. The criteria of influenza are defined as the diagnosis of International Classification of Diseases, Ninth Revision, Clinical Modification (ICD-9-CM) of 487 or 488 or a prescription of Oseltamivir, Peramivir, or Relenza in the index ED visit.

### Definitions of feature variables

We adopted 10 potential risk factors proposed in the previous study for predicting mortality in the older patients with influenza as the feature variables for the ML or DL in this study [4]: (1) tachypnea (respiratory rate > 20/min); (2) severe coma (GCS ≤8, 3) history of hypertension; (4) history of CAD; (5) history of malignancy; (6) bedridden; (7) leukocytosis (WBC > 12,000 cells/mm); (8) bandemia (> 10% band cells); (9) anemia (hemoglobin < 12 mg/dL); and (10) elevated CRP (> 10 mg/dL).

In addition, we also recruited age, sex, vital signs, and past histories of hypertension (ICD-9: 401–405), diabetes (ICD-9-CM: 250), COPD (ICD-9-CM: 496), CAD (ICD-9-CM: 410–414), stroke (ICD-9: 436–438), malignancy (ICD-9: 140–208), congestive heart failure (CHF, ICD-9-CM: 428), dementia (ICD-9: 290), bedridden, feeding with a nasogastric tube, and nursing home resident, laboratory data including white blood cell count (WBC), bandemia, hemoglobin, platelet, serum creatinine, CRP, procalcitonin, glucose, Na, K, GOT, and GPT for this study. The patients who did not have a record of subsequent follow-up were excluded. Missing laboratory data were treated as the normal values (i.e., respiratory rate: 12/min, GCS: 15, WBC: 7000 cells/mm, band form: 0%, hemoglobin: 12 mg/dL, and CRP: 2.5 mg/dL).

### Outcome measurements

The outcome measurements were binary coded as the follows: (1) hospitalization; (2) complications with pneumonia (ICD-9-CM: 480–486): (3) complications with sepsis or septic shock (ICD-9-CM: 038, 790.7, 995.91, 995.91, 785.52); (4) admitted to intensive care unit (ICU); and (5) in-hospital mortality.

### Ethical statement

The present study was approved and granted permission to access the raw data by the institutional review board in the Chi Mei Medical Center. Because this study is retrospective and it contains de-identified information, informed consent from the participants was waived. The waiver does not affect the rights and welfare of the participants.

### Data processing, model comparison, and application in the HIS

First, we extracted, transformed, and validated the data from the HIS into a data mart. Missing and ambiguous data were carefully processed at this step. Second, we randomly split the data to two dataset (70%/30%) and used the synthetic minority oversampling technique (SMOTE) to enlarge the first dataset (70%) as training dataset because of imbalanced outcome samples. The second dataset (30%) is used as testing dataset without any resampling. Third, according the optimal modeling result with testing dataset, we compared accuracy, sensitivity, specificity, positive predictive value, negative predictive value, and the area under the curve (AUC) among the analyses of the random forest, logistic regression, K-nearest neighbors (KNN), support vector machine (SVM), light gradient boosting machine (LightGBM), multilayer perceptron (MLP, a kind of DL), and Extreme Gradient Boosting (XGBoost). In this step, we conducted grid search with hyper-parameters for each algorithm to obtain the optimal models (hyper-parameter ranges for each algorithm were summarized in Supplementary Table 1). Then, we selected the best algorithm to develop the prediction model for each outcome. Fourth, we deployed the model in the AI web service and integrated it with the HIS in the ED. After two-months of pilot testing and validating, we launched the prediction application in the HIS to assist physicians for decision making in real time.

### Patient and public involvement

Patients and the public were not be involved in this study.

## Results

In total, we recruited 5508 older ED patients into the present study. The mean ± standard deviation (SD) of age was 76.61 ± 7.44 years old, and the female proportion was 50.67% (Table 1). The proportion of the three age subgroups were young elderly (43.06%), moderately elderly (40.56%), and old elderly (16.38%). The mean ± SD of respiratory rate and GCS were 19.16 ± 3.94 breaths/min and 14.41 ± 1.84, respectively. The histories of ED patients were hypertension (56.05%), CAD (19.64%), malignancy (14.32%), and bedridden (31.94%). The mean ± SD of WBC, hemoglobin, and CRP were 8670.00 ± 4220.00 cells/mm^3^, 12.42 ± 1.95 mg/dL, and 42.06 ± 50.98 mg/dL, respectively. The proportions of patient outcomes were hospitalization (47.33%), complications with pneumonia (37.71%), complications with sepsis or septic shock (5.57%), admitted to ICU (1.07%), and in-hospital mortality (2.20%).

**Table 1:** Characteristics of older ED patients with influenza in this study

Variable	Total patients (*n* = 5508)
Age (years)	76.61 ± 7.44
Age subgroup (%)	
Young elderly (65–74)	43.06
Moderately elderly (75–84)	40.56
Old elderly (≥85)	16.38
Sex, %	
Female	50.67
Male	49.33
Triage vital signs	
GCS	14.41 ± 1.84
SBP (mm Hg)	142.88 ± 32.77
Heart rate (beats/min)	93.38 ± 24.24
Respiratory rate (breaths/min)	19.16 ± 3.94
Body temperature (°C)	37.53 ± 6.64
Past histories (%)	
Hypertension	56.05
Diabetes	32.37
COPD	12.87
CAD	19.64
CVA	18.77
Malignancy	14.32
CHF	11.27
Dementia	10.62
Bedridden	31.94
Laboratory data	
WBC (cells/mm^3^)	8670.00 ± 4220.00
Bandemia (%)	4.10 ± 5.24
Hemoglobin (mg/dL)	12.42 ± 1.95
Platelet (10^3^/mm^3^)	187.36 ± 72.39
Creatinine (mg/dL)	1.29 ± 1.52
hs-CRP (mg/dL)	42.06 ± 50.98
Sodium (mEq/L)	134.68 ± 4.86
Potassium (mmol/L)	3.76 ± 0.52
GOT (U/L)	51.55 ± 172.64
GPT (U/L)	31.79 ± 64.43
Outcomes (%)	
Hospitalization	47.33
Pneumonia	37.71
Sepsis or septic shock	5.57
ICU admission	1.07
In-hospital mortality	2.20

Data are presented as mean ± SD or percent. *ED* Emergency department; *GCS* Glasgow coma scale; *SBP* Systolic blood pressure; *COPD* Chronic obstructive pulmonary disease; *CAD* Coronary artery disease; *CVA* Cerebrovascular accident; *CHF* Congestive heart failure; *WBC* White blood cell count; *hs-CRP* High sensitivity C-reactive protein; *GOT* Glutamic oxaloacetic transaminase; *GPT* Glutamate pyruvate transaminase; *ICU* Intensive care unit; *SD* Standard deviation

Comparisons of predictive accuracies among the random forest, logistic regression, KNN, SVM, LightGBM, MLP, and XGBoost revealed that the random forest model had the best AUC for hospitalization, pneumonia, and sepsis or septic shock than did other models in the testing dataset (Table 2 and Supplementary Fig. 1). The XGBoost had the best AUC for ICU admission (0.902) and logistic regression had the best AUC for in-hospital mortality (0.889). Table 3 summarized the best AUC for each outcome in the testing dataset, which was adopted for building the prediction model in further. Feature importance according to a random forest, logistic regression, LightGBM, and XGBoost for predicting the five outcomes was also reported (Supplementary Fig. 2).

**Table 2:** Comparisons of predictive accuracies among random forest, logistic regression, KNN, SVM, LightGBM, MLP, and XGBoost in the outcomes of testing dataset of older ED patients with influenza

Outcomes and predictive models	Accuracy	Sensitivity	Specificity	PPV	NPV	AUC
Hospitalization						
Random forest	0.769	0.744	0.791	0.762	0.775	0.840
Logistic regression	0.737	0.751	0.726	0.711	0.764	0.799
KNN	0.736	0.737	0.736	0.715	0.757	0.790
SVM	0.750	0.751	0.749	0.728	0.770	0.840
LightGBM	0.748	0.714	0.780	0.744	0.752	0.823
MLP	0.733	0.702	0.760	0.724	0.740	0.806
XGBoost	0.721	0.705	0.736	0.706	0.735	0.800
Pneumonia						
Random forest	0.679	0.681	0.679	0.562	0.778	0.765
Logistic regression	0.662	0.661	0.662	0.542	0.764	0.709
KNN	0.645	0.700	0.613	0.522	0.771	0.683
SVM	0.657	0.700	0.631	0.534	0.777	0.733
LightGBM	0.653	0.700	0.625	0.530	0.775	0.724
MLP	0.660	0.660	0.660	0.540	0.762	0.688
XGBoost	0.674	0.700	0.658	0.553	0.784	0.744
Sepsis or septic shock						
Random forest	0.795	0.750	0.798	0.179	0.982	0.857
Logistic regression	0.799	0.750	0.801	0.182	0.982	0.832
KNN	0.714	0.750	0.712	0.133	0.980	0.785
SVM	0.707	0.750	0.705	0.130	0.980	0.806
LightGBM	0.739	0.739	0.739	0.143	0.980	0.822
MLP	0.730	0.728	0.730	0.137	0.979	0.761
XGBoost	0.744	0.739	0.744	0.146	0.980	0.811
ICU admission						
Random forest	0.860	0.722	0.862	0.054	0.996	0.885
Logistic regression	0.720	0.778	0.719	0.030	0.997	0.867
KNN	0.607	0.611	0.607	0.017	0.993	0.622
SVM	0.768	0.778	0.768	0.036	0.997	0.778
LightGBM	0.809	0.722	0.810	0.040	0.996	0.874
MLP	0.629	0.611	0.629	0.018	0.993	0.649
XGBoost	0.912	0.722	0.914	0.085	0.997	0.902
In-hospital mortality						
Random forest	0.792	0.806	0.792	0.079	0.995	0.875
Logistic regression	0.816	0.806	0.816	0.089	0.995	0.889
KNN	0.652	0.639	0.652	0.039	0.988	0.663
SVM	0.789	0.722	0.791	0.071	0.992	0.762
LightGBM	0.769	0.722	0.770	0.065	0.992	0.844
MLP	0.675	0.667	0.675	0.044	0.989	0.728
XGBoost	0.751	0.806	0.750	0.067	0.994	0.858

*KNN* K-nearest neighbors; *SVM* Support vector machine; *LightGBM* Light gradient boosting machine; *MLP* Multilayer perceptron, *XGBoost* Extreme Gradient Boosting; *ED* Emergency department; *PPV* Positive predictive value; *NPV* Negative predictive value; *AUC* Area under the curve

**Table 3:** Evaluation report using the best model with the SMOTE preprocessing algorithm on the outcomes of testing dataset of older ED patients with influenza

Outcome	Number	Negative outcome	Positive outcome	Accuracy	Sensitivity	Specificity	PPV	NPV	AUC
Hospitalization (random forest)	5508	2901	2607	0.769	0.744	0.791	0.762	0.775	0.840
Pneumonia (random forest)	5508	3431	2077	0.679	0.681	0.679	0.562	0.778	0.765
Sepsis or septic shock (random forest)	5508	5201	307	0.795	0.750	0.798	0.179	0.982	0.857
ICU admission (XGBoost)	5508	5449	59	0.912	0.722	0.914	0.085	0.997	0.902
In-hospital mortality (logistic regression)	5508	5387	121	0.816	0.806	0.816	0.089	0.995	0.889

*SMOTE* Synthetic minority oversampling technique; *ED* Emergency department; *PPV* Positive predictive value; *NPV* Negative predictive value; *AUC* Area under the curve; *ICU* Intensive care unit; *XGBoost* Extreme Gradient Boosting

We applied the best algorithm for predicting outcomes in older ED patients in the HIS to assist decision making in real time. An AI button was set up in the HIS of the ED (Supplementary Fig. 3). When the clinician presses the AI button, the AI application automatically catch the feature variables from the HIS and pops up a screen of the prediction result within 1 sec (Supplementary Fig. 4). The prediction result shows a personalized prediction for hospitalization, complications with pneumonia, complications with sepsis or septic shock, admitted to ICU, and in-hospital mortality. Using five-level Likert, a mean of 4.6 was responded by 101 times of use, which indicates that the AI prediction is useful for the clinicians.

## Discussion

The present study revealed that the random forest had the best AUC for predicting hospitalization, pneumonia, and sepsis or septic shock, XGBoost had the best AUC for predicting ICU admission, and logistic regression had the best AUC for predicting in-hospital mortality in older ED patients with influenza. The predictions are very fast, in real time, and actionable, which provide prognostic information to assist in decision making, including disposition and outcome explanation.

Using AI prediction for assisting decision-making is an appealing idea [7]. Because of the increased availability of EMRs and advancement of computer performance and algorithm, AI prediction based on the medical big data becomes a promising way for healthcare [8]. In recent years, the rapid progression of cloud and IoT (internet of things) by healthcare monitor and wearable sensor networks also greatly support the development of real-time AI prediction [8]. Therefore, the AI-based tools, which are designed to improve diagnosis, care planning, and outcome will be incorporated into healthcare services in the near future [9]. Many regulations about AI use in healthcare need to be developed, including establishment of normative standard, evaluation guidelines, and monitoring and reporting systems [9].

The adopted feature variables in this study, including comorbidities and abnormal vital signs and laboratory data, are the risk factors for poor outcomes. The more feature variables, the poorer outcome in the result of AI prediction.

The random forest is superior to the traditional model (i.e., logistic regression) for developing CDR in predicting hospitalization, pneumonia, sepsis or septic shock, and ICU admission. One possible reason for the lower predictive accuracies of logistic regression is that it lacks external validation [5]. Traditional CDRs, including the GID score, are typically developed by gathering data at one or more hospitals, and then using both to derive and validate a model from a chosen set of predictors. The developed CDRs are then used in other hospitals, different from the original study hospital [10]. A recent study reviewed 127 new prediction models and showed that external independent validation was uncommon in predictive models [11]. Predictive performance in external validation tends to be worse than the original study [11]. In contrast to the GID score derived from other hospitals, we used local real-world big data in multi-centers to make predictions about the local population, which improves accuracy over the traditionally derived model. The variables used in the present study are structured data from the local EMR without being subjected to ambiguous clinical definitions or biases of data collection.

The random forest model is an ensemble learning method for classification and regression [12, 13]. It combines many binary decision trees, which are built by several bootstrapped learning samples, and chooses a subset of variables randomly at each node [12, 13]. Each tree in the random forest will vote for some input x, then the voting majority of trees will determine the output of the classifier [14]. The random forest can use a large number of trees in the ensemble to handle high dimensional data [14]. The random forest is a common method adopted for predicting outcomes and selecting predictors in the ED. A study about predicting in-hospital mortality in ED patients with sepsis revealed that the AUC of the random forest was 0.86, superior to the CART (classification and regression tree) model (0.69); logistic regression model (0.76); CURB-65 (Confusion, Urea, Respiratory rate, Blood pressure plus age ≥ 65 years old) (0.73); MEDS (mortality in emergency department sepsis) (0.71); and mREMS (modified rapid emergency medicine score) (0.72) [5]. A study used the random forest to select the most relevant variables for major adverse cardiac events in ED patients with chest pain [12]. They found that the selection predictor by the random forest is promising in discovering a few relevant and significant predictors [12].

The SMOTE adopted in the present study is the most common and effective method of oversampling for adjusting imbalanced data [15]. SMOTE solves the problems of both high-class skew and high sparsity and works in the “feature space” rather than “data space” [16]. By taking each minority class sample and the K-nearest neighbors, SMOTE creates synthetic samples for effectively forcing the decision region of the minority class to become more general [16]. Without duplicating the data, SMOTE increases the data space and amplifies the features of the minority class [16]. Studies with SMOTE preprocessing in health care are also acceptable [17, 18].

According to our literature review, the present study has the strength of being the first real-time prediction application in the HIS using ML for older ED patients with influenza. The limitations are as follows. First, interpretability and inferences about variables are the problems of ML. Second, we did not compare the predictive accuracy between this model and the physician’s judgment. Further studies about this issue, as well as the impact of this model, are warranted. Third, variable selection was not conducted in this study. We decided to adopt 10 potential risk factors proposed in the previous study for increasing the explainability for AI models. In the future, including as many variables as possible and reducing the number by running proper variable selection algorithms are needed. Fourth, the application may not be generalized to other hospitals because it needs building an infrastructure to make real-time predictive analytics a reality.

## Conclusions

We developed the first real-time prediction application in the HIS for predicting outcomes in older ED patients with influenza using a big data-driven and machine learning approach. This real-time prediction is a promising way to assist the physician’s decision making and explanations to patients and their families. Further studies about the predictive accuracy between this model and both the physician’s judgment, impact of the application, and including as many variables as possible and reducing the number by running proper variable selection algorithms are needed.

## Supplementary Information

**Additional file 1.**

## Data Availability

The datasets used during the current study are available from the corresponding author on reasonable request.

## Abbreviations

ED: Emergency department
ML: Machine learning
DL: Deep learning
COPD: Chronic obstructive pulmonary disease
GID: Geriatric influenza death
GCS: Glasgow coma scale
CAD: Coronary artery disease
CRP: C-Reactive protein
CI: Confidence interval
CDR: Clinical decision rule
AI: Artificial intelligence
EMRs: Electronic medical records
HIS: Hospital information system
ICD-9-CM: International classification of diseases, ninth revision, clinical modification
ICU: Intensive care unit
SMOTE: Synthetic minority oversampling technique
AUC: Area under the curve
KNN: K-nearest neighbors
SVM: Support vector machine
LightGBM: Light gradient boosting machine
MLP: Multilayer perceptron
XGBoost: Extreme gradient boosting
SD: Standard deviation
CART: Classification and regression tree
CURB-65: Confusion, urea, respiratory rate, blood pressure plus age ≥ 65
MEDS: Mortality in emergency department sepsis
mREMS: Modified rapid emergency medicine score
