# Supplementary material for: Predicting outcomes in older ED patients with influenza in real time using a big data-driven and machine learning approach to the hospital information system
Source: BMC Geriatr. 2021 Apr 27;21:280. doi: 10.1186/s12877-021-02229-3 (PMC8077903; doi:10.1186/s12877-021-02229-3)
Supplement: Supplementary file 1 — Additional file 1. [file 12877_2021_2229_MOESM1_ESM.docx]

**Supplementary Table 1.** Hyper-parameters range for experiments.

| Method and  Hyper-parameter | Outcomes -Hospitalization |  | Outcomes -  Pneumonia |  | Outcomes -  Sepsis or septic shock |  | Outcomes -  ICU admission |  | Outcomes -  In-hospital mortality |
| --- | --- | --- | --- | --- | --- | --- | --- | --- | --- |
|  | Values |  | Values |  | Values |  | Values |  | Values |
| Logistic regression |  |  |  |  |  |  |  |  |  |
| penalty | l1, l2 |  | l1, l2 |  | l1, l2 |  | l1, l2 |  | l1, l2 |
| C | 0.001, 0.01, 0.09, 1, 1.2, 5, 15, 30, 100 |  | 0.001, 0.01, 1, 1.5, 5, 15, 30, 100, 1000 |  | 0.001, 0.01, 1, 1.5, 10, 15, 30, 100, 1000 |  | 0.001, 0.01, 0.09, 1, 1.5, 15, 30, 100, 1000 |  | 0.001, 0.01, 1.5, 15, 30, 100, 1000 |
| max_iter | 100, 500, 1000, 2500 |  | 100, 500, 1000, 1500 |  | 100, 500, 1000, 1500 |  | 100, 500, 1000, 1500 |  | 100, 500, 1000, 1500 |
| Random forest |  |  |  |  |  |  |  |  |  |
| n_estimators | 500, 750, 950, 1000, 2000 |  | 500, 750, 950, 1000, 2000 |  | 500, 750, 950, 1000, 2000 |  | 250, 300, 500, 700,750, 1000 |  | 250, 300, 500, 700, 950 |
| max_depth | 15, 30, 50, 90, 150 |  | 15, 30, 50, 100 |  | 15, 30, 45, 50, 100 |  | 15, 30, 50, 90 |  | 15, 30, 50, 90 |
| min_samples_split | 2, 4, 6, 10, 15 |  | 2, 6, 10, 15 |  | 2, 6, 10, 15 |  | 2, 4, 6, 10, 15 |  | 2, 6, 10, 15 |
| max_features | auto, sqrt, 0.5, 1.0, 2.5 |  | auto, sqrt, 0.5, 1.0, 2.5 |  | auto, sqrt, 0.5, 1.0, 2.5 |  | auto, sqrt, 0.5, 1.0, 2.5 |  | auto, sqrt, 0.5, 1.0, 2.5 |
| KNN |  |  |  |  |  |  |  |  |  |
| n_neighbors | range(1,20) |  | range(1,20) |  | range(1,20) |  | range(1,20) |  | range(1,20) |
| weights | uniform, distance |  | uniform, distance |  | uniform, distance |  | uniform, distance |  | uniform, distance |
| algorithm | auto, ball_tree, kd_tree, brute |  | auto, ball_tree, kd_tree, brute |  | auto, ball_tree, kd_tree, brute |  | auto, ball_tree, kd_tree, brute |  | auto, ball_tree, kd_tree, brute |
| leaf_size | range(1,5) |  | range(1,5) |  | range(1,5) |  | range(1,5) |  | range(1,5) |
| SVM |  |  |  |  |  |  |  |  |  |
| kernel | rbf, linear |  | rbf, linear |  | rbf, linear |  | rbf, linear |  | rbf, linear |
| gamma | scale, 1e-2, 1e-3, 1e-4 |  | scale, 1e-2, 1e-3, 1e-4 |  | scale, 1e-2, 1e-3, 1e-4 |  | scale, 1e-2, 1e-3, 1e-4 |  | scale, 1e-2, 1e-3, 1e-4 |
| C | 1, 10, 100, 500, 1000, 2500, 5000 |  | 1, 10, 100, 500, 1000, 2500, 5000 |  | 1, 10, 100, 500, 1000, 1500 |  | 1, 5, 10, 150, 250, 300 |  | 20, 50, 150, 200 |
| decision_function_shape | ovo, ovr |  | ovo, ovr |  | ovo, ovr |  | ovo, ovr |  | ovo, ovr |
| shrinking | True, False |  | True, False |  | True, False |  | True, False |  | True, False |
| LightGBM |  |  |  |  |  |  |  |  |  |
| learning_rate | 1e-4, 1e-3, 1e-2 |  | 1e-4, 1e-3, 1e-2 |  | 1e-4, 1e-3, 1e-2 |  | 1e-4, 1e-3, 1e-2 |  | 1e-4, 1e-3, 1e-2 |
| num_iterations | 75, 150, 200, 500 |  | 100, 200, 500 |  | 100, 200, 500, 750, 1000 |  | 150, 300, 500, 750 |  | 150, 250, 500, 750 |
| max_depth | 12, 15, 30, 50 |  | 15, 30, 50 |  | 15, 30, 50 |  | 15, 30, 50 |  | 15, 30, 50 |
| random_state | 8, 16, 42 |  | 8, 16, 42 |  | 8, 16, 42 |  | 8, 16, 42 |  | 8, 16, 42 |
| XGBoost |  |  |  |  |  |  |  |  |  |
| learning_rate | 1e-4, 1e-3, 1e-2 |  | 1e-4, 1e-3, 1e-2 |  | 1e-4, 1e-3, 1e-2 |  | 1e-4, 1e-3, 1e-2 |  | 1e-4, 1e-3, 1e-2 |
| gamma | 1e-2, 1e-3, 1e-4, 1e-5 |  | 1e-2, 1e-3, 1e-4, 1e-5 |  | 1e-2, 1e-3, 1e-4, 1e-5 |  | 1e-2, 1e-3, 1e-4 |  | 1e-2, 1e-3, 1e-4 |
| num_iterations | 200, 500, 750, 900 |  | 200, 500, 750, 820,900 |  | 200, 500, 750, 900, 1000 |  | 100, 200, 250, 350, 500 |  | 150, 200, 350, 550 |
| max_depth | 15, 25, 30, 50 |  | 15, 25, 30, 50 |  | 15, 25, 30, 50 |  | 15, 30, 50 |  | 15, 30, 50 |
| num_parallel_tree | 2, 5, 15 |  | 2, 5, 15 |  | 2, 5, 15 |  | 2, 5, 10 |  | 2, 5, 8, 10 |
| MLP Classifier |  |  |  |  |  |  |  |  |  |
| hidden_layer_sizes | (400,300,100,70),  (350,150), (250,100), (220,175,60), (200,100), (90,45) |  | (400,300,100,70),  (400,180,100),  (350, 180, 70),  (350, 150, 75),  (300, 150, 75), |  | (400,250,110,70),  (350, 150, 75),  (300, 150, 75),  (220, 175, 60) |  | (400,250,110,70),  (400,300,100,70),  (350, 150, 75),  (300, 150, 75),  (250, 110, 75) |  | (400, 300, 100, 70),  (400, 280, 100, 70),  (350, 150, 75),  (300, 150, 75),  (250, 110, 75) |
| earning_rate_init | 1e-3, 1e-2 |  | 1e-3, 1e-2 |  | 1e-3, 1e-2 |  | 1e-3, 1e-2 |  | 1e-3, 1e-2 |
| early_stopping | True, False |  | True, False |  | True, False |  | True, False |  | True, False |

The hyper-parameters that are not described in this table are set to the default values used in the scikit-learn library (Grid search with 10-fold cross-validation for hyper-parameters tuning for each algorithm was conducted for obtaining optimal model). ICU, intensive care unit; KNN, K-nearest neighbors; SVM, support vector machine; LightGBM, light gradient boosting machine; MLP, multilayer perceptron; XGBoost, Extreme Gradient Boosting.

**Supplementary Figure 1.** AUCs according to seven algorithms for predicting hospitalization, pneumonia, sepsis or septic shock, ICU admission, and in-hospital mortality in older ED patients with influenza.

| **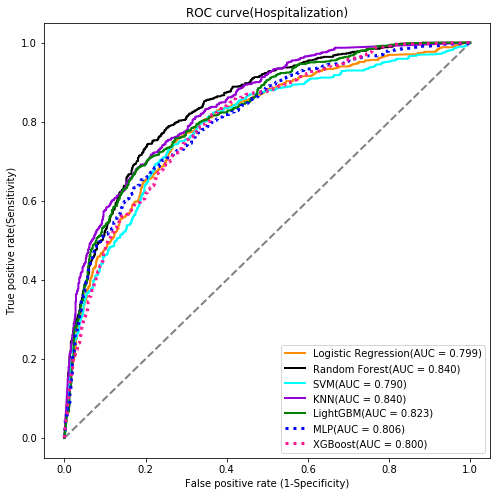** | **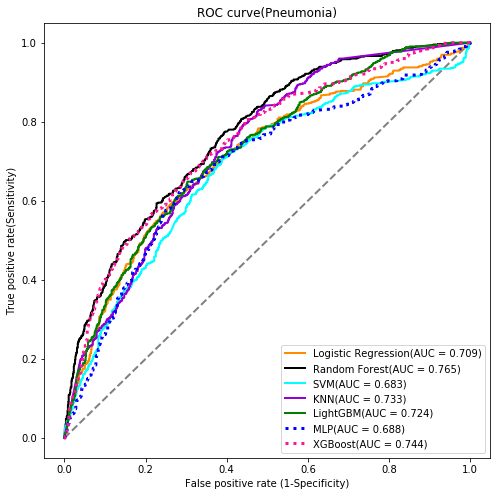** | **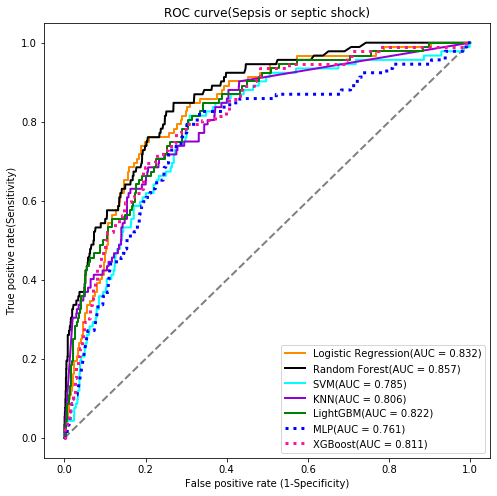** |
| --- | --- | --- |
| **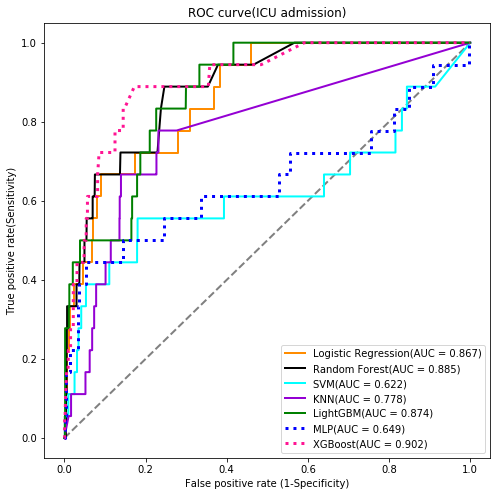** | **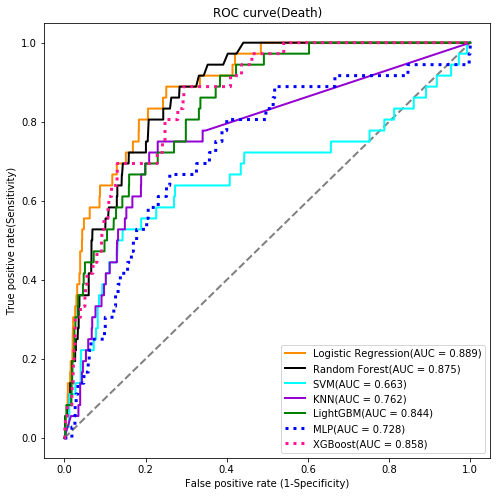** |  |

AUC, area under the curve, ICU, intensive care unit; ED, emergency department.

**Supplementary Figure 2.** Feature importance for predicting hospitalization, pneumonia, sepsis or septic shock, ICU admission, and in-hospital mortality in older ED patients with influenza.

Feature importance -Hospitalization

| 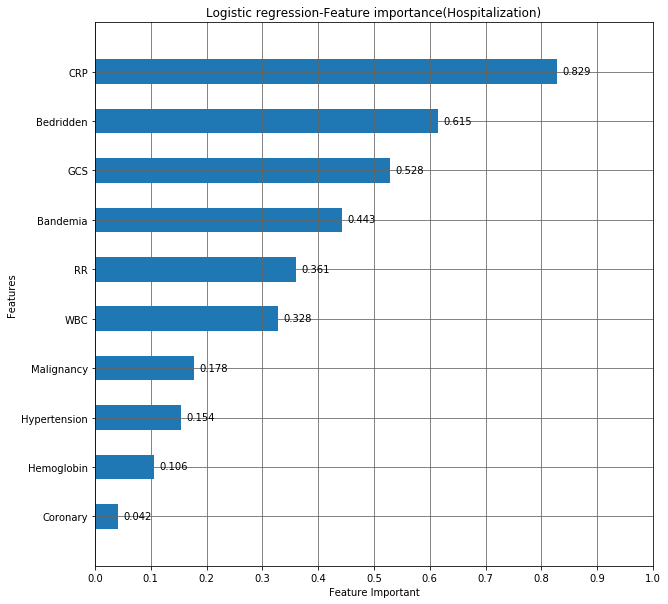 | 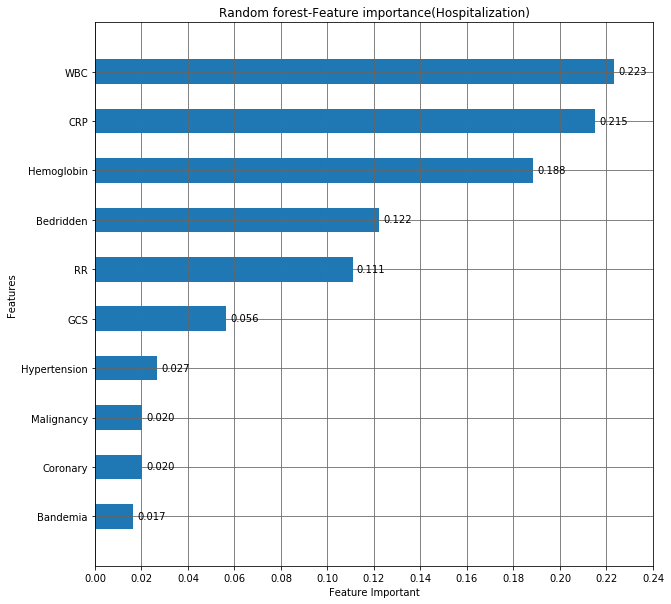 | 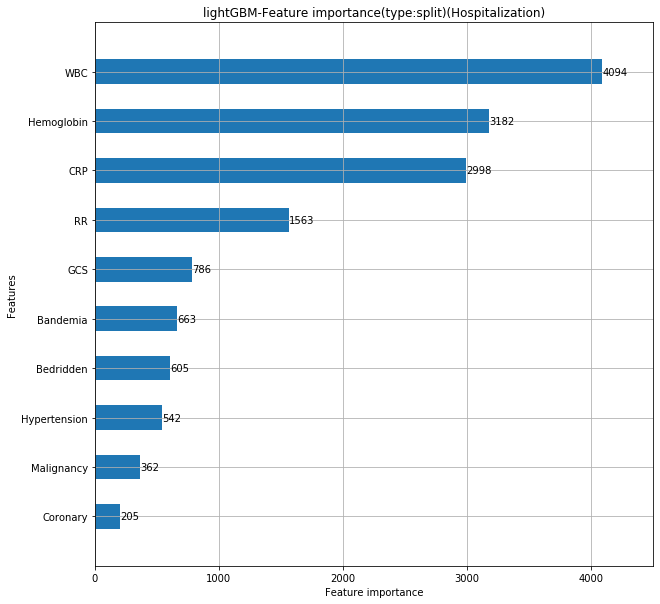 |
| --- | --- | --- |
| 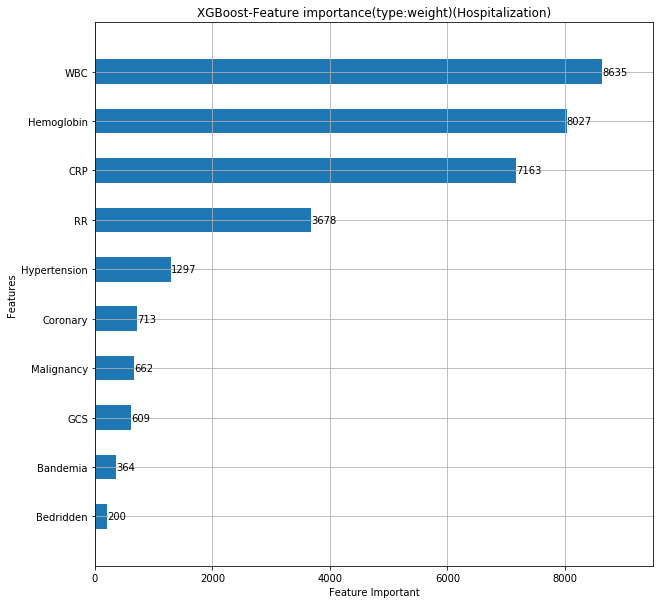 |  |  |

Feature importance - Pneumonia

| 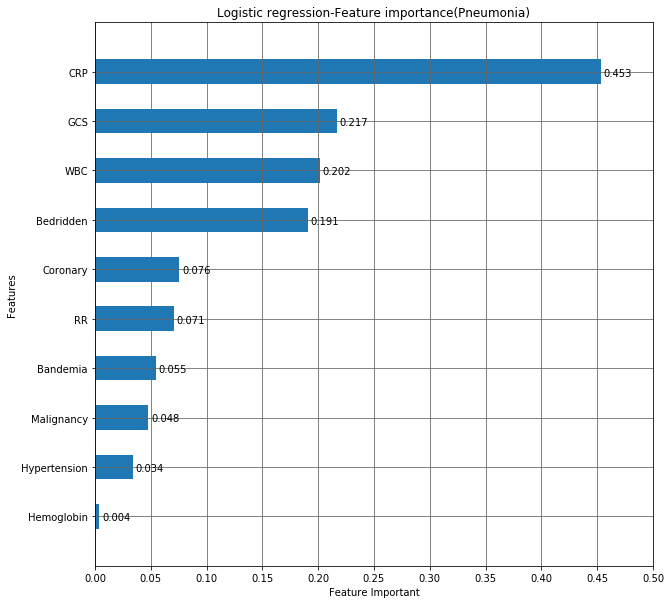 | 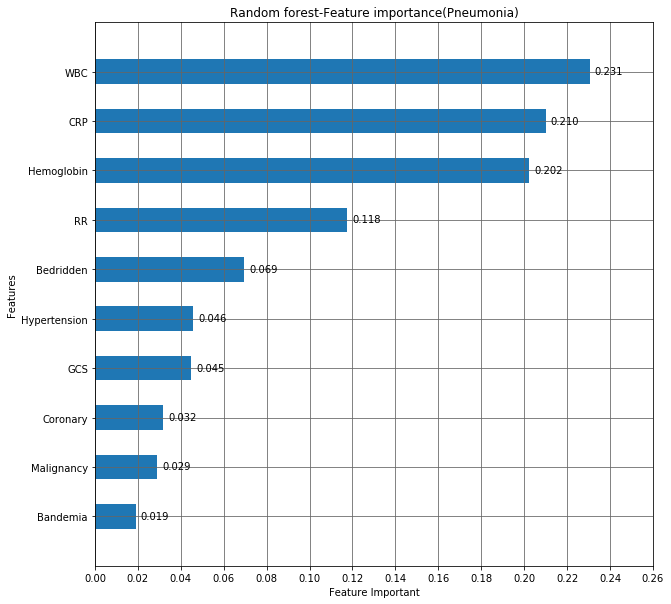 | 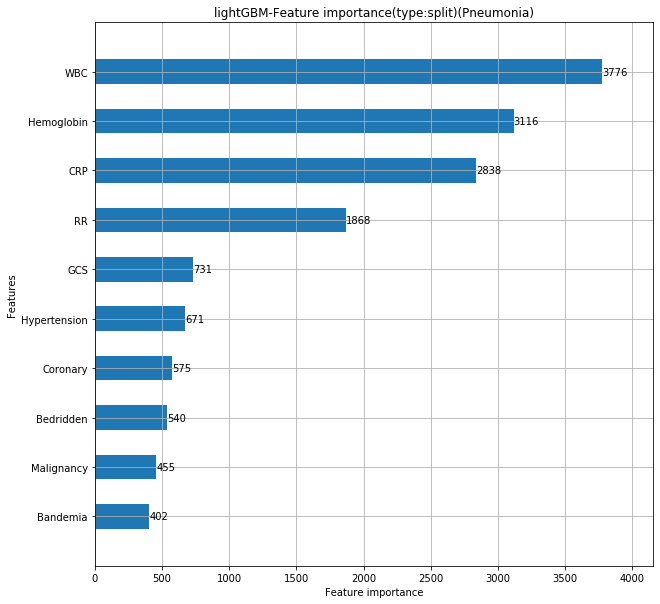 |
| --- | --- | --- |
| 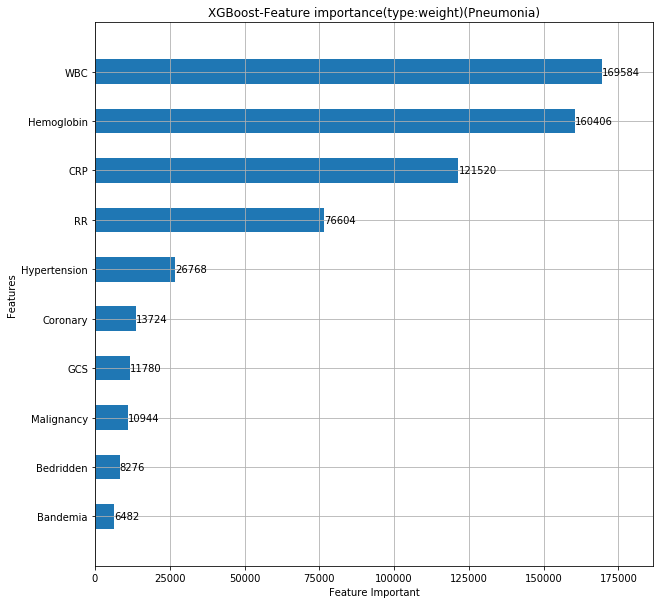 |  |  |

Feature importance - Sepsis or septic shock

| **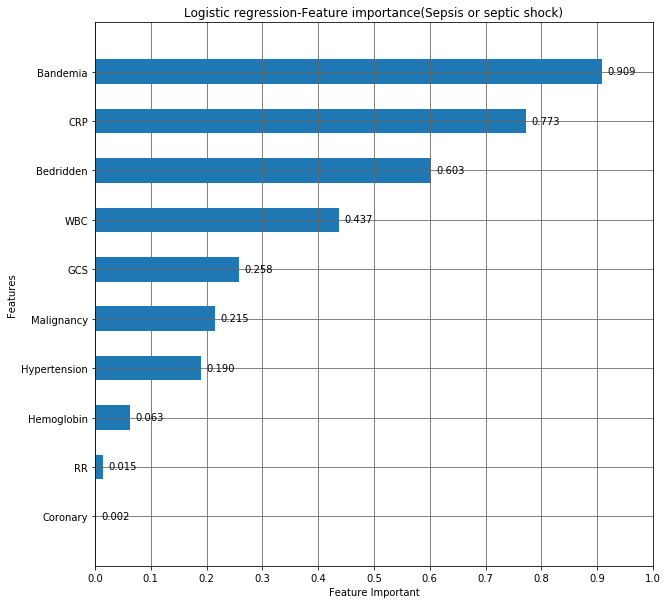** | **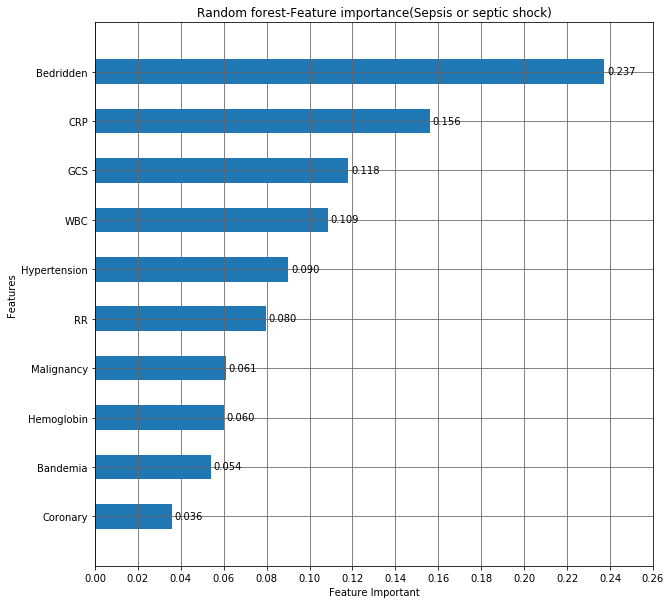** | **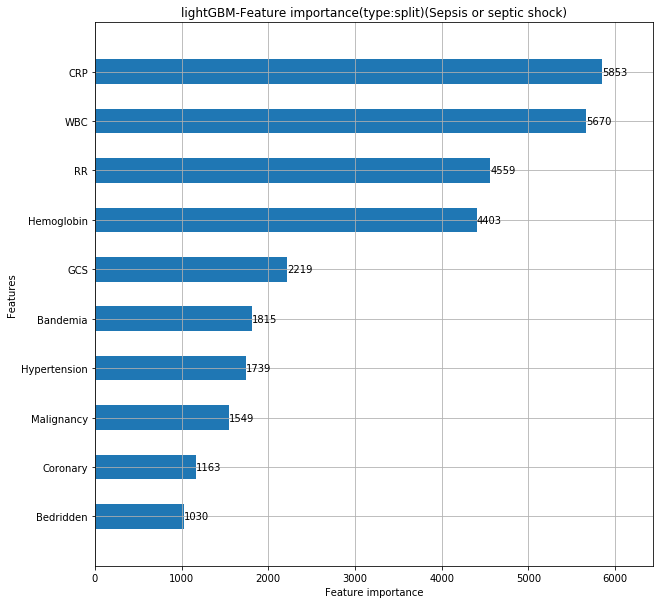** |
| --- | --- | --- |
| **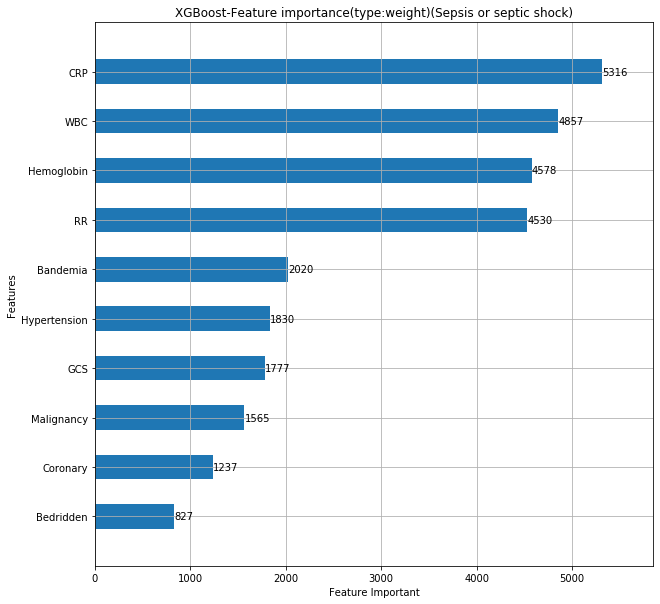** |  |  |

Feature importance - ICU admission

| **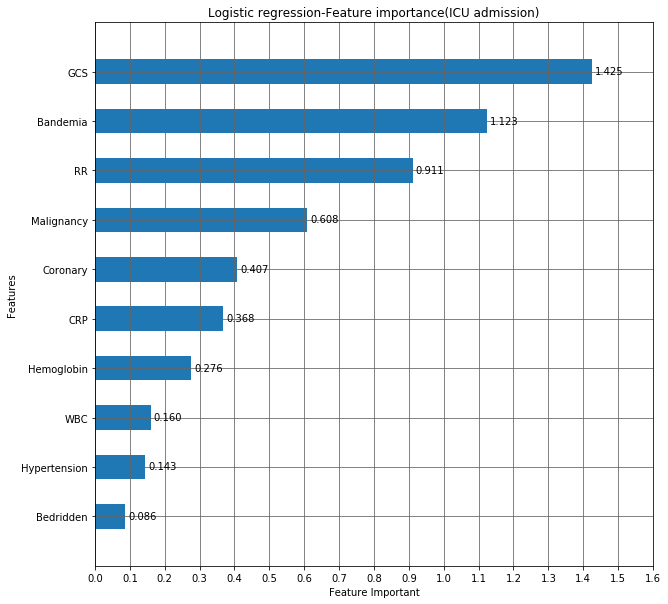** | **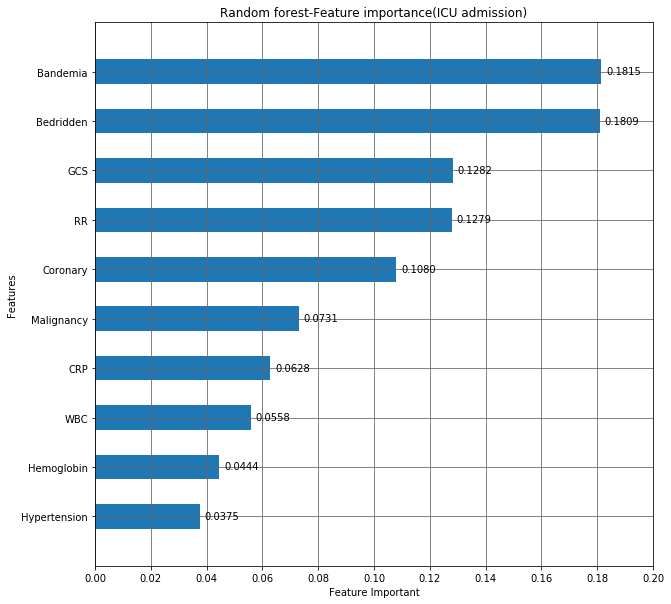** | **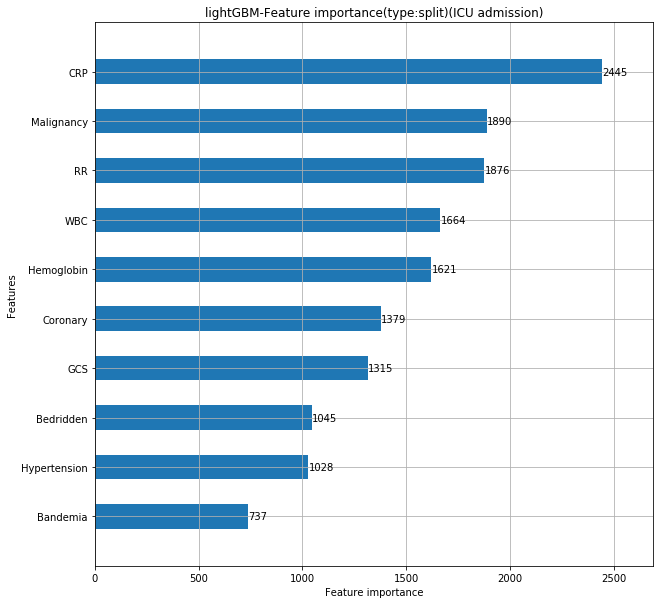** |
| --- | --- | --- |
| **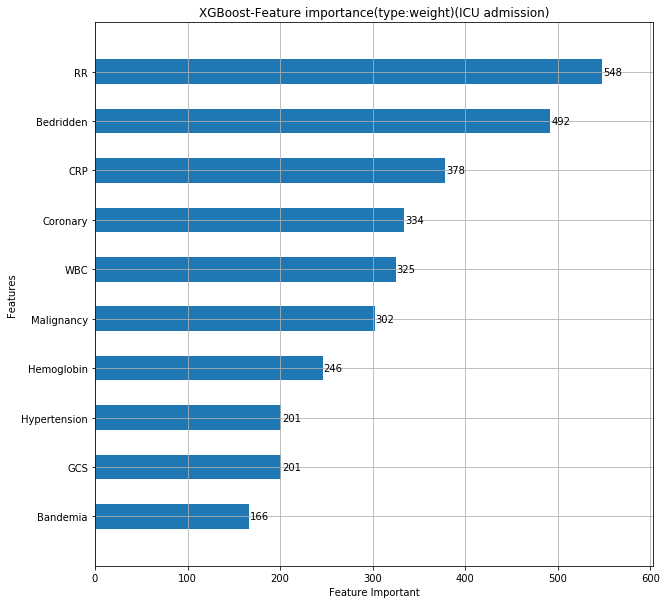** |  |  |

Feature importance – in-hospital mortality

| **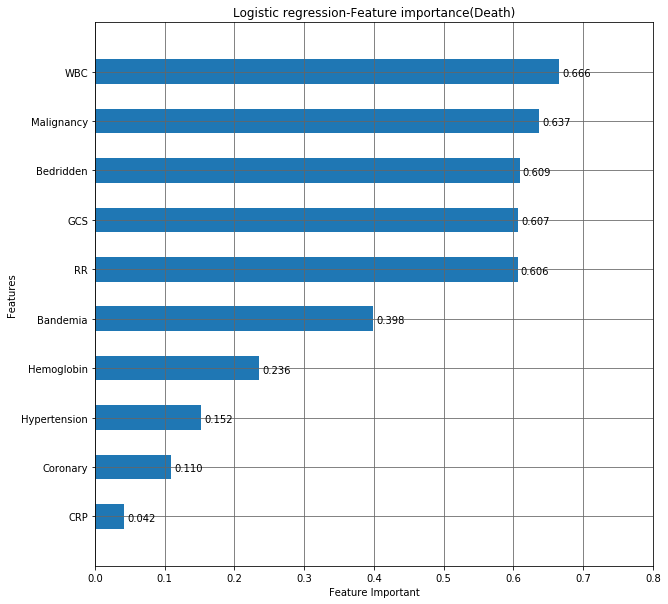** | **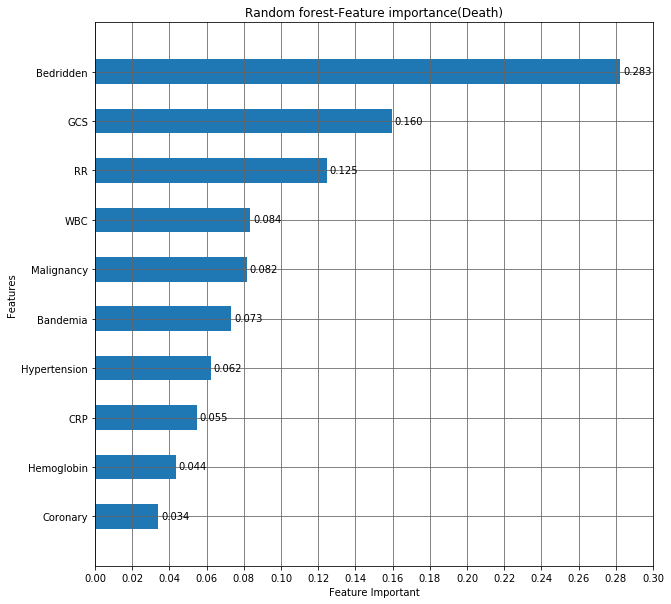** | **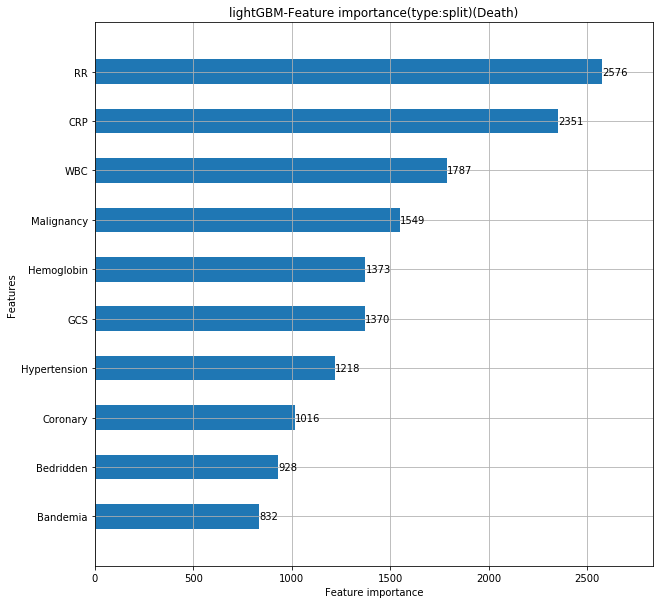** |
| --- | --- | --- |
| **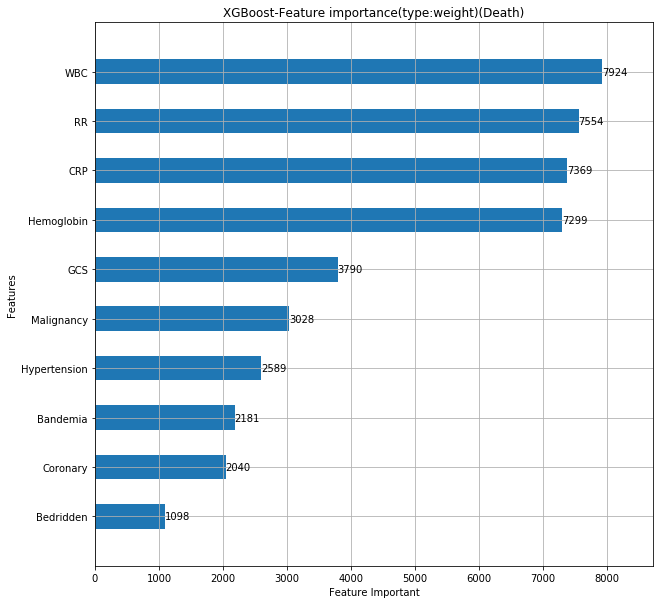** |  |  |

ICU, intensive care unit; ED, emergency department.

**Supplementary Figure 3.** An AI button was set up in the HIS of the ED.


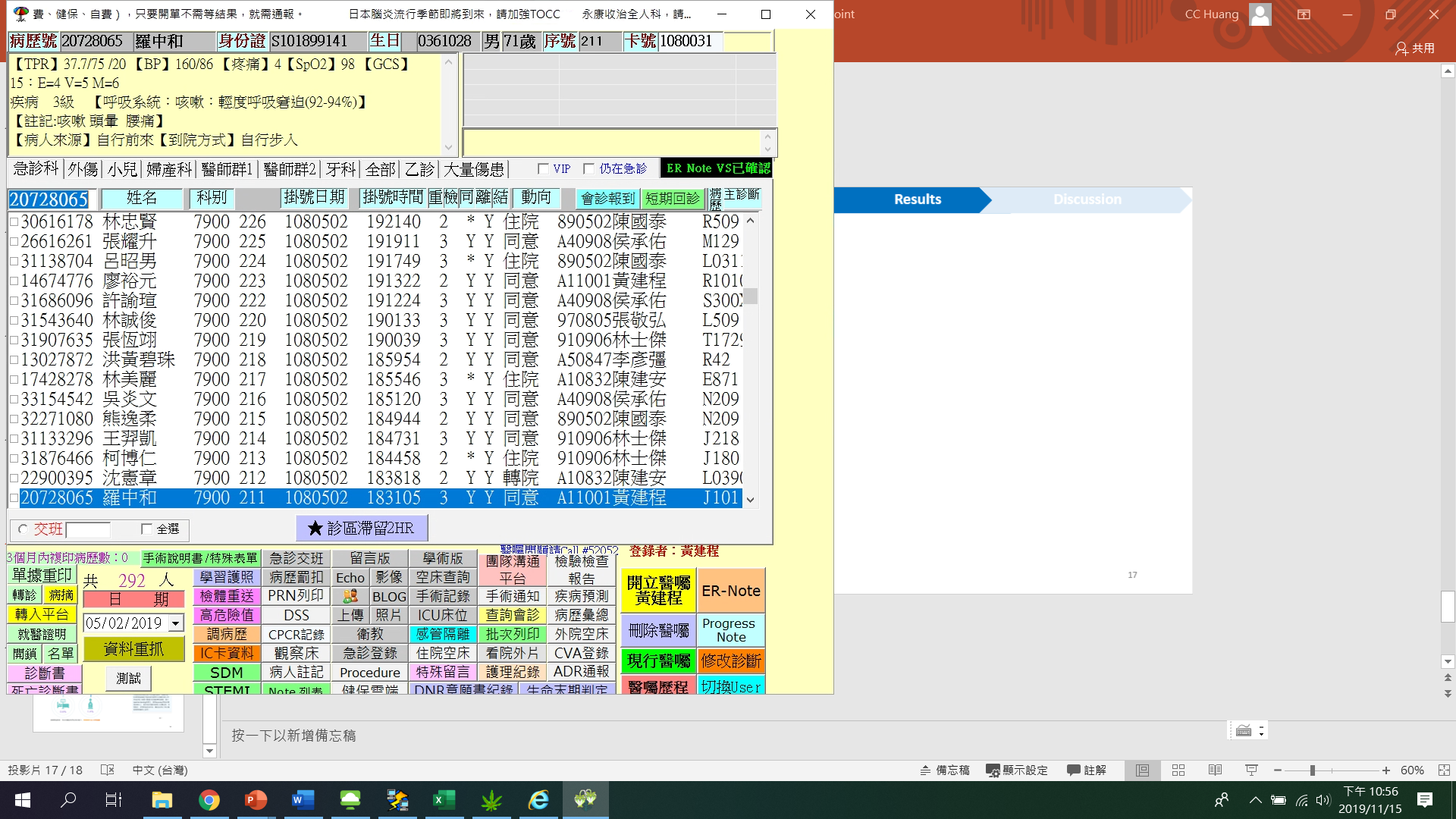


AI button

AI, artificial intelligence; HIS, hospital information system; ED, emergency department.

**Supplementary Figure 4.** Screenshot of the real-time AI prediction model in the HIS for predicting five outcomes in older ED patients with influenza.

AI prediction for adverse outcomes in older patients with influenza

Feature variable of the patient

Pneumonia


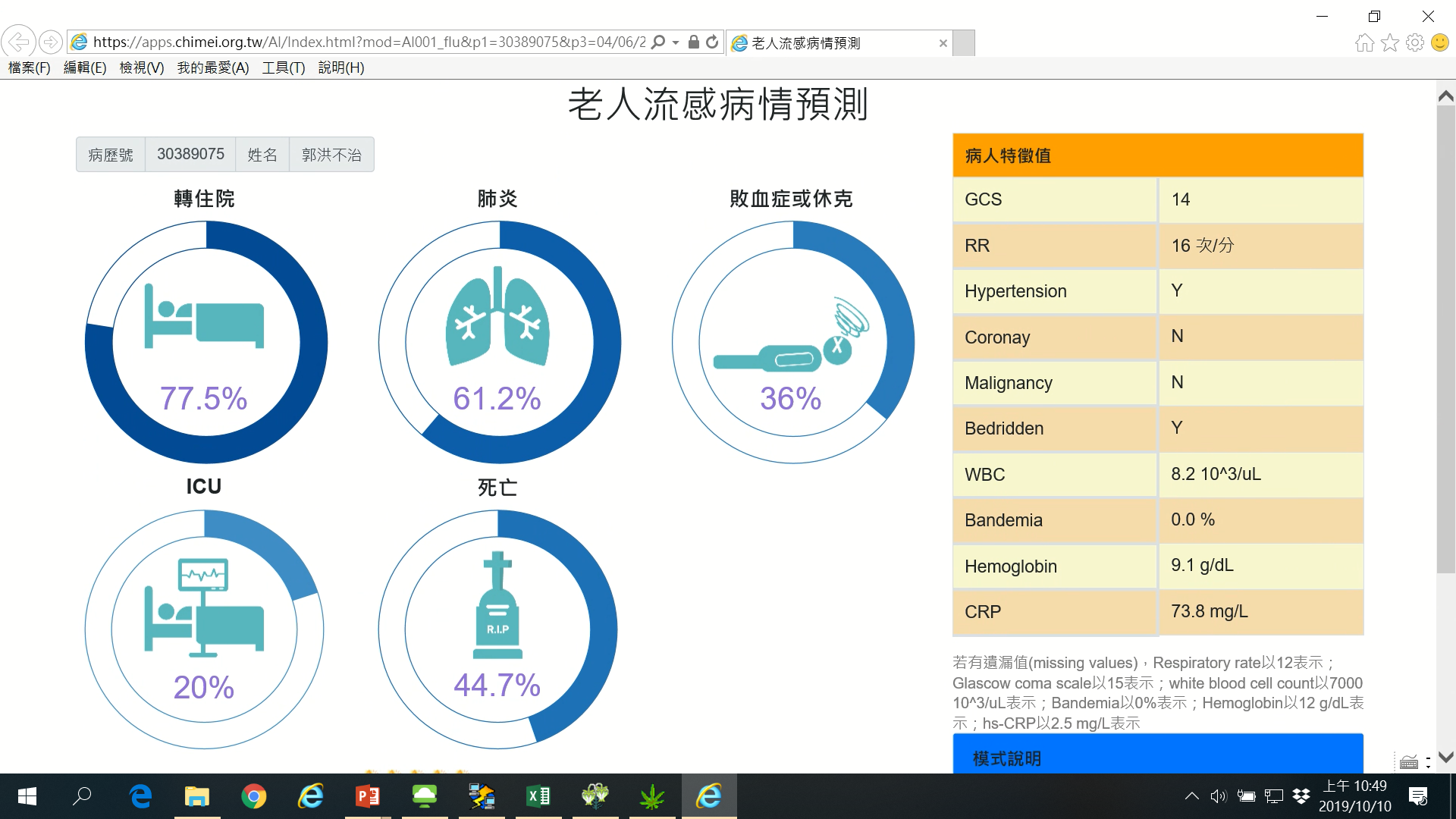


In-hospital mortality

Sepsis or septic shock

Admitted to ICU

Hospitalization

AI, artificial intelligence; HIS, hospital information system; ED, emergency department; ICU, intensive care unit.
